# The anti-diarrhea activity of red algae-originated sulphated polysaccharides on ETEC-K88 infected mice

**DOI:** 10.1039/c8ra09247h

**Published:** 2019-01-18

**Authors:** Bo Liu, Qing-Mei Liu, Gui-Ling Li, Le-Chang Sun, Yuan-Yuan Gao, Ya-Fen Zhang, Hong Liu, Min-Jie Cao, Guang-Ming Liu

**Affiliations:** College of Food and Biological Engineering, Xiamen Key Laboratory of Marine Functional Food, Fujian Provincial Engineering Technology Research Center of Marine Functional Food, Fujian Collaborative Innovation Center for Exploitation and Utilization of Marine Biological Resources, Jimei University 43 Yindou Road Xiamen 361021 Fujian P. R. China gmliu@jmu.edu.cn +86-592-6180470 +86-592-6180378

## Abstract

Polysaccharides from red algae *Porphyra haitanensis* and *Gracilaria lemaneiformis* possess various bioactive functions, however, their anti-diarrhea activity remains incompletely defined. In the current study, sulphated polysaccharides were extracted by high pressure treatment plus ethanol precipitation from these two algae, and named PHSP_(hp)_ and GLSP_(hp)_, respectively. PHSP_(hp)_ and GLSP_(hp)_ showed decreased viscosity and molecular weight. Meanwhile, they have a certain immunomodulatory effect on wound healing and migration of RAW264.7 cells. Moreover, they significantly increased the secretion of pro-inflammatory cytokines tumor necrosis factor-α (TNF-α) and interleukin-6 (IL-6). A BALB/c model infected by enterotoxigenic *Escherichia coli* (ETEC)-K88 was also established to evaluate the anti-diarrhea activity of PHSP_(hp)_ and GLSP_(hp)_. The results showed that PHSP_(hp)_ and GLSP_(hp)_ were able to alleviate mice diarrhea symptoms. Meanwhile, they inhibited the release of pro-inflammatory cytokines and suppressed the secretion of immunoglobulin A *via* reducing the population of B cells. In addition, the nitroblue tetrazolium levels of mouse serum were decreased. Taken together, PHSP_(hp)_ and GLSP_(hp)_ alleviated the inflammatory response of ETEC-K88-induced diarrhea through both specific and non-specific immunity. Sulphated polysaccharides from red algae may be used as functional food components for remitting diarrhea.

## Abstract 2

Polysaccharides from red algae *Porphyra haitanensis* and *Gracilaria lemaneiformis* possess various bioactive functions, however, their anti-diarrhea activity remains incompletely defined.

## Graphical abstract

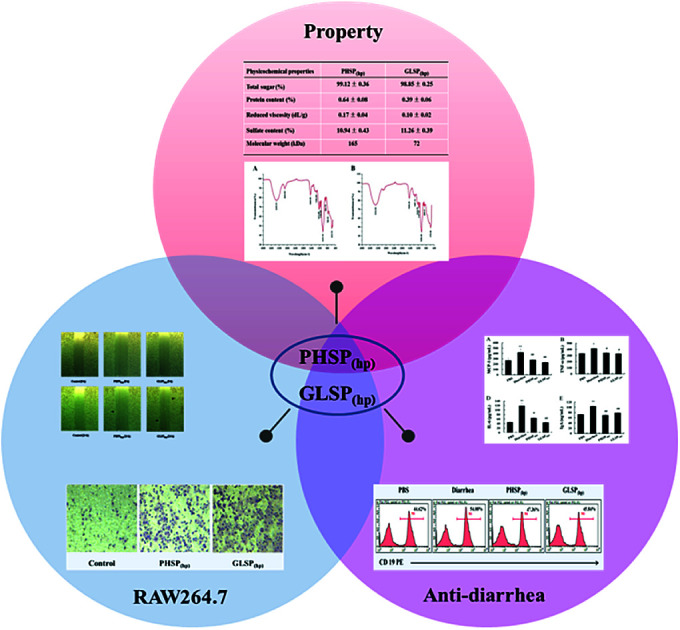

## Introduction

Diarrhea is a digestive disorder disease generally characterized by abnormally frequent defaecation (three times or more per day), and/or blood and poorly fecal matter in feces, as well as electrolyte imbalance.^1^ According to epidemiological analysis, diarrhea is one of the most common diseases with global incidence greater than one billion per year.^2^ Although nowadays its mortality rate has been reduced due to improved medical care, it still remained the second leading lethal cause for children under age five, particularly in the developing countries, as reported in 2012.^3,4^ Among various types of infectious diarrhea, bacterial infection can cause a cute diarrhea which is characterized by fever, vomiting, abdominal pain, nausea and anorexia.^5^ The most common bacterial dysentery pathogens include *Escherichia coli*, *Salmonella*, *Shigella* and *Staphylococcus aureus*.^6^ Some strains of *E. coli* cause infections in the gastrointestinal tract system, while others may lead to non-gastrointestinal infections such as bacteremia, hospital pneumonia and neonatal meningitis.^7^

The enterotoxigenic *Escherichia coli* (ETEC) expresses one or more fimbrial adhesins, including F4 (K88), F5 (K99), F6 (987P), F7 (F41) and F18, that tightly bind to glycoprotein receptors on the enterocyte brush border to enable bacteria colonization in the small intestine. The bacteria then secrete heat-labile enterotoxins and/or heat-stable enterotoxins that alter the tight junction integrity and disrupt the paracellular passages of ions, solutes and water, leading to diarrhea.^8–10^

Current main therapies for diarrhea consist of oral rehydration solutions (ORS) and medications such as antibiotics. ORS is the first-line treatment for diarrhea worldwide, however, it is unable to reduce diarrhea's duration and severity.^11^ Antibiotic therapy was considered as one of the most effective diarrhea treatments in the past. However, it might develop antibiotic resistance in bacteria, which counteracts diarrhea treatment.^11^ Up to date, there has been no vaccine available against ETEC-induced diarrhea of children or travelers. Therefore treatment with bioactive molecule from marine resource with therapeutic potential for infectious diarrhea has gained growing research interest.^12^

Among bioactive components, the polysaccharides from seaweed are of special interest due to their great biological activities. It has been reported that sulphated polysaccharides from red algae have different biological activities, such as anti-inflammatory,^13^ anti-microbial,^14,15^ antiviral,^16^ anti-cancer,^17^ and antioxidant^18^ bioactivities. *Porphyra haitanensis* (*P. haitanensis*) and *Gracilaria lemaneiformis* (*G. lemaneiformis*) are the two major species of red algae cultured in China, with annual product of 115 875 and 270 149 tons, respectively in 2016.^19^ Our previous work showed that sulphated polysaccharides extracted from *P. haitanensis* and *G. lemaneiformis* exhibited a certain anti-allergic activity.^20,21^ However, the anti-diarrhea activity of red algae-originated sulphated polysaccharides is rarely reported.

This study aims to investigate the immunomodulatory and anti-diarrhea activity of sulphated polysaccharides isolated from *P. haitanensis* and *G. lemaneiformis* in RAW264.7 cells and a mouse model with ETEC-K88-induced diarrhea. The present research may provide insights into future exploration for anti-diarrhea functional foods and/or their feed.

## Materials and methods

### Reagents

Fetal bovine serum (FBS) was purchased from Gibco (Grand Island, NY, USA). DMEM was obtained from HyClone Co. (Logan, UT, USA). The enzyme-linked immunosorbent assay (ELISA) kits for immunoglobulin A (IgA), tumor necrosis factor-α (TNF-α), interferon-γ (IFN-γ), interleukin-6 (IL-6) and monocyte chemotactic protein 1 (MCP-1) were purchased from R&D Systems (Minneapolis, MN, USA). The antibodies, including APC-conjugated CD3, PerCP-Cy5.5-conjugated CD4 and PE-conjugated CD19, were obtained from Biolegend (San Diego, CA, USA). Other chemicals were purchased from Sigma-Aldrich (St. Louis, MO, USA) and are all analytical grade.

### Extraction of the polysaccharides from red algae

The red algae *P. haitanensis* and *G. lemaneiformis* were purchased from a local market of Dadeng Island (Xiamen, Fujian, China). Polysaccharide extraction was performed according to the method of Shi *et al.* with slight modifications.^20^ Briefly, the grated dry algae tissue (10 g) is mixed with 400 mL of distilled water and autoclaved under high pressure at 121 °C for 5 h. Being filtered with a nylon membrane, the polysaccharides were precipitated with 4 volumes of ethanol and kept at 4 °C for 24 h. The filtrate was filtered again with a nylon membrane, naturally dried at room temperature, and re-dissolved in a small amount of water followed by freeze-dry under −50 °C. The sulphated polysaccharides extracted from *P. haitanensis* and *G. lemaneiformis* with high pressure were named PHSP_(hp)_ and GLSP_(hp)_, respectively. The yield of polysaccharides was calculated as the following formula: polysaccharides recovery rate (%) = mass (g) of dried material obtained/mass (g) of original dried microalgae × 100.

### Characterization of the polysaccharide

The viscosity of polysaccharide was determined using an Ubbelohde viscometer. Protein content of the polysaccharide fraction was measured by the Bradford method as previously described.^22^ The total carbohydrate and sulfate content in PHSP_(hp)_ and GLSP_(hp)_ were determined as previously described.^20^ The molecular weight distribution of polysaccharides was determined by high performance gel-permeation chromatography (HPGPC) (Agilent-1100, Santa Clara, U.S.A.) equipped with a ZORBAX Eclipse XDB-C18 column (250 × 4.6 mm^2^, column temperature 30 °C). The Fourier transform infrared spectra of polysaccharide was analyzed with a Fourier transformed infrared spectrometer (FT-IR) (Vector-22, Bruker, Switzerland) in the wave number range of 4000–400 cm^−1^ using the KBr-disk method.

### RAW264.7 cell culture, wound healing assay and transwell migration assay

The murine monocyte-macrophage cell line RAW264.7 from Wuhan Beinglay Biotech Co. (Wuhan, Hubei, China) was grown in DMEM medium supplemented with 10% FBS, 100 U mL^−1^ penicillin and 100 μg mL^−1^ streptomycin at 37 °C in an incubator with a humidified atmosphere of 5% CO_2_.

The wound healing assay was carried out to investigate the immunomodulation activity of PHSP_(hp)_ and GLSP_(hp)_ on RAW264.7 macrophages in 6-well culture plate.^23^ Briefly, after the cells adhere and the density was greater than 95%, they were scratched by small tips and continued to incubate with 200 μg mL^−1^ PHSP_(hp)_ or GLSP_(hp)_ for 24 h. Images were taken at 0 h and 24 h. The degree of healing (three wells per set) at 24 h was determined as compared to that of cell at 0 h.

The migration assay was carried out as described before.^24^ RAW264.7 cells (1 × 10^5^) were added to the upper chamber of transwell inserts (8 μm pores, 6.5 mm polycarbonate membranes, Costar, Corning, NY, USA) that were placed in the 24-well culture plate. Six hundred microliter of DMEM complete medium together with 200 μg mL^−1^ PHSP_(hp)_ or GLSP_(hp)_ was added to the lower chamber. Twenty four hours after incubation, the cells on the topside of upper chamber were removed with a cotton swab, followed by washing with PBS to remove the residual medium. Cells adherent the opposite surface of the insert was fixed with paraformaldehyde and stained with 0.1% crystal violet for 20 min, and washed 2–3 times with tap water. The cell migration was observed under microscope (three wells per group). Finally, the bound dye was released with 30% glacial acetic acid, and its optical density was measured at 570 nm using an automated ELISA plate reader.

### Measurement of released TNF-α and IL-6

The RAW264.7 cells (1 × 10^5^ cells per well) were seeded in 48-well plates with different concentrations of PHSP_(hp)_ and GLSP_(hp)_ (0, 25, 50, 100 and 200 μg mL^−1^) or lipopolysaccharide (LPS) (2 μg mL^−1^) as a positive control. After 24 h incubation, the culture supernatant was collected and assayed using the ELISA kits for TNF-α and IL-6, following the manufacturer's instructions (R&D, Minneapolis, MN, USA).

### Establishment of ETEC-K88 bacteria induced-diarrhea mouse model

The 6 week-old specific-pathogen-free (SPF) female BALB/c mice were purchased from the Shanghai Laboratory, Animal Center of the Chinese Academy of Sciences (Shanghai, China). Mice were maintained in a SPF environment at 22 ± 1 °C with relative 55 ± 10% humidity. All animal procedures were performed in accordance with the guidelines for care and use of Laboratory Animals of Jimei University and experiments were approved by the Animal Ethics Committee of Jimei University (Xiamen, Fujian, China), SCXK 2012-0005. Mice were fed with standard chows (Beijing Keao Biological Pharmaceutical Co., Ltd, Beijing, China) and water *ad libitum* throughout the experiments, except for fast prior to ETEC-K88 infection.

Standard strains of the Gram-negative enterotoxigenic bacteria *Escherichia coli* (ETEC-K88) freeze-drying powder (bio-10666) was purchased from Beijing Biobw Biotechnology Co., Ltd. (Biobw, Beijing, China). The bacteria were grown in Luria–Bertani (LB) medium, and incubated at 37 °C with shaking for 12 h. Cultures were harvested by centrifugation at 5000*g* for 10 min, and the pellet was washed twice and re-suspended in sterile phosphate buffer saline (PBS). The counting of bacteria was carried out by means of inverted plate and their OD_600_ value.

The 50% lethal dose (LD_50_) of ETEC-K88 was determined using a dose–response method.^25^ Thirty six female BALB/c mice were randomly divided into six groups. Mice were fasted for 12 h before infection, except for access to water *ad libitum*. The next day, mice in different groups were administrated in intraperitoneal injection with approximately 2 × 10^8^, 3 × 10^8^, 4 × 10^8^, 5 × 10^8^, 6 × 10^8^ CFU of ETEC-K88 in PBS at a volume of 200 μL per mouse. The control group was administrated in intraperitoneal injection with sterile PBS alone. Food was then reintroduced *ad libitum*. Animals were observed four times per day for clinical signs and mortality for a period of 72 h. Mice that were still alive 3 days after bacterial challenge were recorded as survivors. The total number of dead mice at each dose level was recorded and the LD_50_ was determined from a dose lethality curve using the Prism 6 statistical software of GraphPad Prism (San Diego, CA, USA).

Forty female BALB/c mice were randomly divided into four groups, including PBS control group, diarrhea group, PHSP_(hp)_ group and GLSP_(hp)_ group. To induce diarrhea, the mice were injected intraperitoneally with 0.2 mL of ETEC-K88 at the level of LD_50_. Three hours later, two groups were orally administered PHSP_(hp)_ and GLSP_(hp)_ (10 mg per mice), respectively.^26,27^ The other two groups were orally administered PBS (200 μL per mice). After that, all doses were given once every 24 h for one week.

The animals were observed 30 min after treatment and periodically during the first 24 h, with special attention given during the first 4 h, and daily thereafter, for a total of 7 days. The symptoms of illness or abnormal behavior were recorded as Leódido described.^28^ Furthermore, the body weight and diarrhea rate of mice were monitored throughout the study period.

### Measurement of the levels of cytokines and IgA in mouse serum

The blood samples were collected from orbital venous plexus 24 h after the last administration and centrifuged at 4000*g* for 10 min. The sera were obtained and the levels of MCP-1, TNF-α, IFN-γ, IL-6 and IgA were measured by ELISA according to the manufacturer's instructions.

### Flow cytometry analysis of T/B cell subpopulation

A small portion of the spleen was used for splenocytes preparation. The splenocytes were freed of red blood cells upon treatment with lysis buffer (Solabio Co., Beijing, China), stained with CD3-APC, CD4-PerCP-Cy5.5 and CD19-PE for 30 min at 4 °C and the T and B lymphocyte subpopulation was analyzed by flow cytometry. A Guava easyCyte 6-2L system were used for the analysis with the GuavaSoft 3.1.1 software (Millipore, MA, USA), following the manufacturer's instructions.

### Determination of nitroblue tetrazolium (NBT) activity

The NBT assay was performed according to Anderson and Siwicki (1995) with slight modification.^29^ The blood (50 μL) was transferred into a glass tube containing equal amount of 0.2% (w/v) NBT solution (prepared in PBS), or equal amount of PBS as the control. The tubes were incubated at room temperature for 30 min before addition of 1.5 mL *N*,*N*-dimethyl formamide (DMF). The tubes were then centrifuged at 3000*g* for 5 min at room temperature. The supernatant was collected and the sample absorbance at 540 nm was determined on an automated ELISA plate reader.

### Statistical analysis

The data were expressed as mean ± standard deviation (SD) and the group difference was analyzed by one-way ANOVA of Duncan test using the SPSS 17.0 software package (IBM, USA), and considered significant with *P* < 0.05. Regarding the animal model assays, samples from individual mouse were processed and analyzed separately. Each experiment was repeated at least 3 times.

## Results

### Polysaccharide extraction and property analysis

**Fig. 1:**
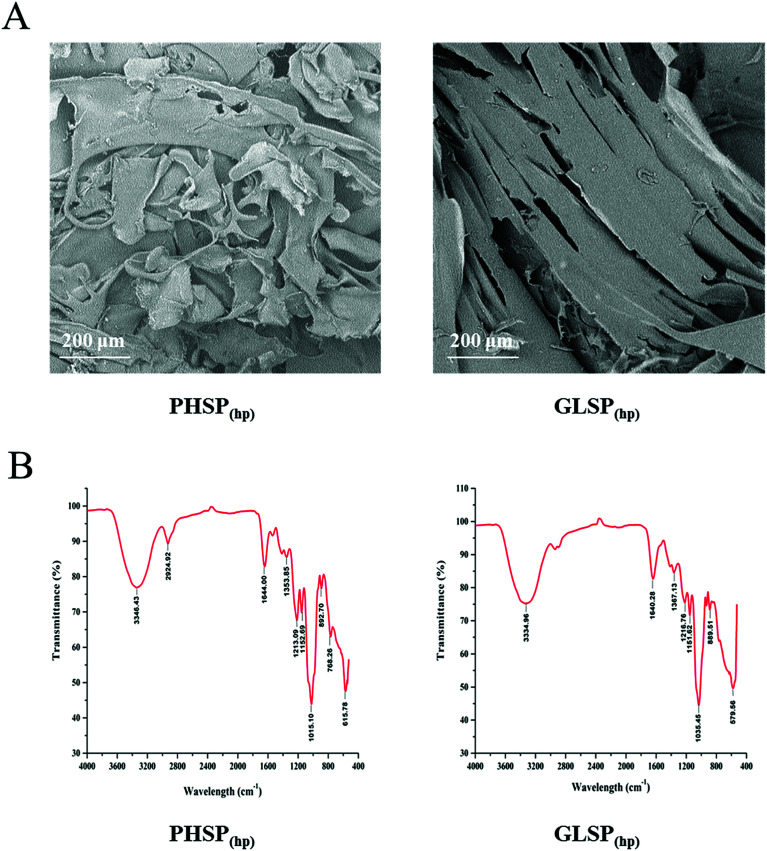
SEM and FT-IR spectrum of PHSP_(hp)_ and GLSP_(hp)_. (A) SEM of PHSP_(hp)_ and GLSP_(hp)_. (B) FT-IR spectra of PHSP_(hp)_ and GLSP_(hp)_.

Using high pressure treatment and ethanol precipitation, 4.11 ± 0.36 g and 2.77 ± 0.19 g of polysaccharides were obtained from 10 g *P. haitanensis* and *G. lemaneiformis*, respectively. The microstructures of polysaccharides were also determined under a scanning electron microscope (SEM), as show in Fig. 1A. The microstructure of PHSP_(hp)_ has an irregular geometrical shape with a certain gap on the surface and loosely superimpose. However, the surface structure of GLSP_(hp)_ was smooth and flat, showing a continuous sheet.

The total sugar content of the extracted substance was above 98% and few protein residues were detected in the polysaccharides fraction. As shown in Table 1, PHSP_(hp)_ and GLSP_(hp)_ have viscosity of 0.17 dL g^−1^ and 0.10 dL g^−1^. The average molecular weight of PHSP_(hp)_ was 165.45 kDa, while GLSP_(hp)_ have two components, 71.87 kDa (accounting for 96.36%) and 1641.95 kDa (3.64%). The FT-IR spectrum showed that PHSP_(hp)_ and GLSP_(hp)_ exhibited absorption peaks at 3330 cm^−1^ and 1640 cm^−1^, representing –OH and C=C group, respectively (Fig. 1B). Furthermore, the absorption peaks at 1216 and 890 cm^−1^ for sulfated components were present. The sulfate content in PHSP_(hp)_ and GLSP_(hp)_ were further determined as 10.94% and 11.26%, respectively.

**Table 1:** Physicochemical properties of PHSP_(hp)_ and GLSP_(hp)_

Physicochemical properties	PHSP_(hp)_	GLSP_(hp)_
Total sugar (%)	99.12 ± 0.36	98.85 ± 0.25
Protein content (%)	0.64 ± 0.08	0.39 ± 0.06
Viscosity (dL g^−1^)	0.17 ± 0.04	0.10 ± 0.02
Sulfate content (%)	10.94 ± 0.43	11.26 ± 0.39
Average molecular weight (kDa)^a^	165.45	71.87

a Represents the major component (>96.0%).

### Effects of PHSP_(hp)_ and GLSP_(hp)_ on RAW264.7 wound healing and migration

**Fig. 2:**
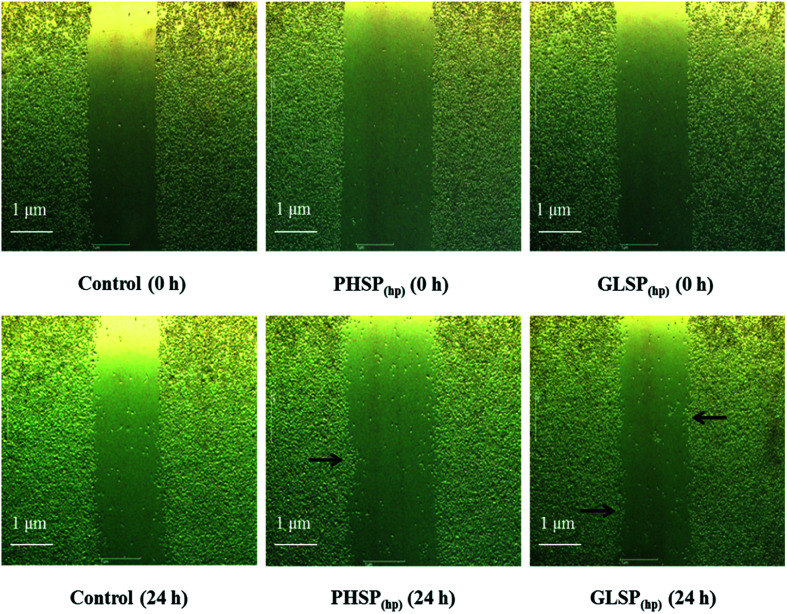
The influence of PHSP_(hp)_ and GLSP_(hp)_ on wound healing of RAW264.7 cells (100×, 24 h). The cells were scratched, and incubated with PBS (as the control group), PHSP_(hp)_ or GLSP_(hp)_ followed by a 24 h-recovery period. Microscopic bright field pictures were taken immediately after the scratch and the precise coordinates were reassessed 24 h after incubation.

To investigate the effects of PHSP_(hp)_ and GLSP_(hp)_ on the inflammatory cells, their wound healing stimulating activity on RAW264.7 cells were evaluated using the scratch assay. Compared with the control, the number of cells in the middle wound area was increased upon treatment with PHSP_(hp)_ or GLSP_(hp)_ for 24 h (Fig. 2). The cells in the marginal area had a tendency to heal in the middle. Therefore PHSP_(hp)_ and GLSP_(hp)_ had effective healing capability for the scratched RAW264.7 cells.

**Fig. 3:**
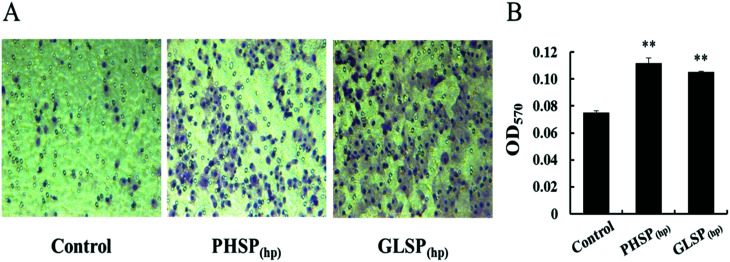
The influence of PHSP_(hp)_ and GLSP_(hp)_ on RAW264.7 migration (100×, 24 h). (A) RAW264.7 cell migration micrograph. (B) The absorbance values (OD_570_) of eluted crystal violet. Data are present as mean ± SD (*n* = 3).

The influence of PHSP_(hp)_ and GLSP_(hp)_ on RAW264.7 migration was also determined. As shown in Fig. 3A, 200 μg mL^−1^ PHSP_(hp)_ and GLSP_(hp)_ significantly promoted the migration of RAW264.7 from the upper chamber of transwell to its bottom side. The number of migrated cells in PHSP_(hp)_ and GLSP_(hp)_ groups were significantly higher than that in the control group (Fig. 3A). The absorbance values (OD_570_) of eluted crystal violet also showed that PHSP_(hp)_ and GLSP_(hp)_ significantly promoted RAW264.7 migration, as shown in Fig. 3B.

### Effects of PHSP_(hp)_ and GLSP_(hp)_ on the production of TNF-α and IL-6 in RAW264.7 cells

**Fig. 4:**
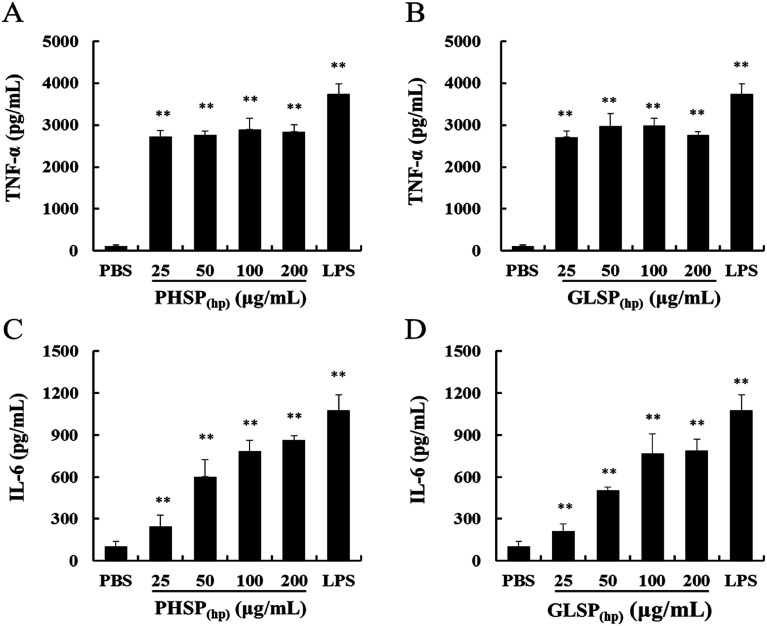
Effects of PHSP_(hp)_ and GLSP_(hp)_ on TNF-α and IL-6 production in RAW264.7 cell supernatant. RAW264.7 cells were incubated with PHSP_(hp)_ and GLSP_(hp)_ (0, 25, 50, 100, 200 μg mL^−1^) for 24 h and the cultural supernatants were collected for ELISA. (A and B) Effects of PHSP_(hp)_ and GLSP_(hp)_ on the secretion of TNF-α. (C and D) Effects of PHSP_(hp)_ and GLSP_(hp)_ on the secretion of IL-6. PBS served as a negative control and 2 μg mL^−1^ LPS served as a positive control. Data are present as mean ± SD (*n* = 3). *P < 0.05 *versus* the PBS group, **P < 0.01 *versus* the PBS group.

The inflammatory factors of RAW264.7 cells were further explored. As shown in Fig. 4, exposure of RAW264.7 cells to PHSP_(hp)_ and GLSP_(hp)_ for 24 h resulted in significant increases in the secretion of TNF-α and IL-6, compared to PBS. The levels of TNF-α were significantly increased to 2897.75 pg mL^−1^ and 2983.13 pg mL^−1^, respectively, in the PHSP_(hp)_ and GLSP_(hp)_ (100 μg mL^−1^) treated group (Fig. 4A and B). However, the secretion level of IL-6 increased in a dose-dependent manner in PHSP_(hp)_ and GLSP_(hp)_ treated RAW264.7 cells (Fig. 4C and D), which were 861.48 pg mL^−1^ and 788.23 pg mL^−1^, respectively, in 200 μg mL^−1^ PHSP_(hp)_ and GLSP_(hp)_ treated cells. These findings suggested that PHSP_(hp)_ and GLSP_(hp)_ regulated the immune system by enhancing the secretion of pro-inflammatory cytokines.

### Alleviation of PHSP_(hp)_ and GLSP_(hp)_ on diarrhea symptoms

**Fig. 5:**
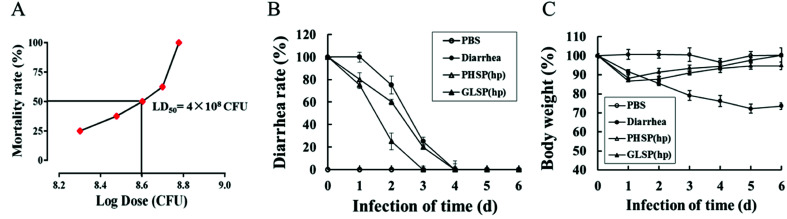
Effects of PHSP_(hp)_ and GLSP_(hp)_ on diarrhea rate and weight changes in ETEC-K88 infected diarrhea mice. (A) Dose lethality curve of ETEC-K88 in BALB/c mice. (B) Variation of diarrhea rate along with infection time. (C) Weight changes along with infection time. Data (A) is present as mean (*n* = 6), data (B and C) are present as mean ± SD (*n* = 10).

To investigate the anti-diarrhea activity of PHSP_(hp)_ and GLSP_(hp)_*in vivo*, an ETEC-K88 induced mice model were established in the present study. The study found that the ETEC-K88 at all dose caused death, and mice of the highest dose group died after two days. The LD_50_ of the ETEC-K88 was determined as 4 × 10^8^ CFU in female BALB/c mice (Fig. 5A).

The mice infected with ETEC-K88 (LD_50_) could suffer diarrhea after 30 min (data not shown), excreting dilute stool like water. Compared with the diarrhea group, the PHSP_(hp)_ and GLSP_(hp)_ treated mice showed reduced infectious diarrhea rate in the first four days (Fig. 5B). On day 4, all the diarrhea mice returned to normal. The weight of mice infected with ETEC-K88 was significantly reduced on the first day. In addition, the weight of diarrhea group mice decreased for 5 days in a row, while the weight of PHSP_(hp)_ or GLSP_(hp)_ treated mice rebounded on the second day (Fig. 5C). These results suggest that PHSP_(hp)_ and GLSP_(hp)_ can reduce diarrhea rate and put on weight to alleviate diarrhea symptoms.

### Effects of PHSP_(hp)_ and GLSP_(hp)_ on cytokines and IgA antibody levels in mice serum

**Fig. 6:**
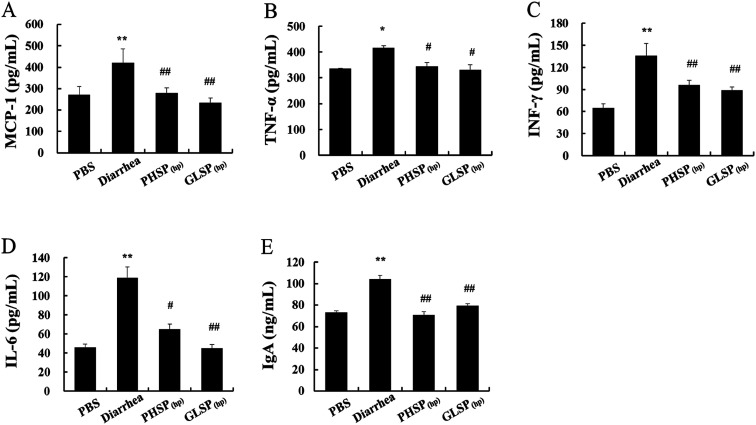
Effects of PHSP_(hp)_ and GLSP_(hp)_ in mice serum cytokines. (A) MCP-1, (B) TNF-α, (C) IFN-γ, (D) IL-6, (E) IgA. ELISA was carried out in different ETEC-K88-infected sample groups. Data are present as mean ± SD (*n* = 3). *P < 0.05 *versus* the PBS group, and **P < 0.01 *versus* the PBS group. ^#^*P* < 0.05 *versus* the diarrhea group, and ^##^*P* < 0.01 *versus* the diarrhea group.

The previous study reported that ETEC-K88-induced mice diarrhea caused inflammatory response with highly secretion of pro-inflammation mediators.^30^ As shown in Fig. 6A–D, the levels of pro-inflammatory cytokines (MCP-1, TNF-α, IFN-γ, IL-6) were all significantly reduced in PHSP_(hp)_ and GLSP_(hp)_ treated mice serum.

The changes in total IgA levels in the serum in different groups were also measured and the data were shown in Fig. 6E. The serum IgA concentration was 104.21 ng mL^−1^ in the diarrhea group, which was significantly higher than that of the control PBS group at 73.04 ng mL^−1^ (*P* < 0.01), indicating increased inflammatory response. Moreover, serum IgA levels in the PHSP_(hp)_ and GLSP_(hp)_ groups were decreased to 70.54 ng mL^−1^ and 79.20 ng mL^−1^, respectively. These results suggested that PHSP_(hp)_ and GLSP_(hp)_ remitted inflammatory response in ETEC-K88 induced mice diarrhea.

### Effects of PHSP_(hp)_ and GLSP_(hp)_ on T/B cell subpopulation of mice splenocytes

**Fig. 7:**
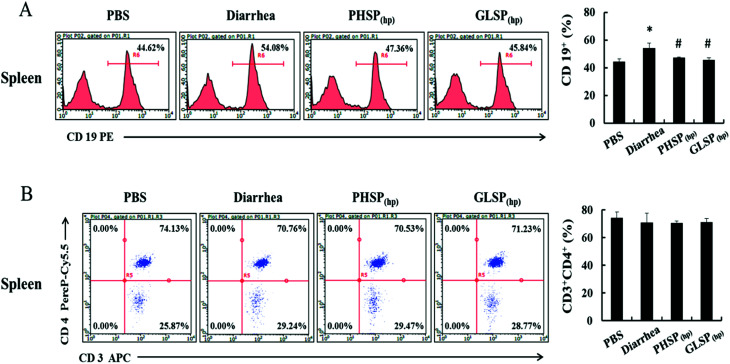
Effects of PHSP_(hp)_ and GLSP_(hp)_ on lymphocyte from mice splenocytes. The infected mice were fed with PHSP_(hp)_ and GLSP_(hp)_ for 7 days before they were sacrificed and the splenocytes were obtained. Cells of 1 × 10^6^ were mixed with CD3-APC, CD4-PercP-Cy5.5 and CD19-PE for 30 min at 4 °C, before subject to the flow cytometry. (A) The histogram of B cell FACS analysis. (B) The scatter diagrams of Th cell FACS analysis.

Considering the suppression effects of PHSP_(hp)_ and GLSP_(hp)_ on serum IgA, the classification of mice spleen lymphocytes was further performed by flow cytometry. Compared with PBS group, the CD19^+^ B cells population in the diarrhea group was increased about 10% to 54.08%, indicating that the B cell subpopulation was increased upon ETEC-K88 infection (Fig. 7A). And the CD19^+^ B cells population were 47.36% and 45.84% in PHSP_(hp)_ and GLSP_(hp)_ (10 mg d^−1^) treatment mice spleen, respectively, suggesting that PHSP_(hp)_ and GLSP_(hp)_ (10 mg d^−1^) down-regulated B cell population. Moreover, PHSP_(hp)_ and GLSP_(hp)_ showed no effect on CD3^+^CD4^+^ Th cell population (Fig. 7B). Taken together, it was tempting to speculate that PHSP_(hp)_ and GLSP_(hp)_ reduce B cells subpopulation, which suppress IgA expression in mice serum and contribute to the anti-inflammatory activities in ETEC-K88-induced mice diarrhea.

### Effects of PHSP_(hp)_ and GLSP_(hp)_ on the NBT activity in mice serum

The NBT assay was used to determine the neutrophil activity as nonspecific immune factor.^25^ As shown in Table 2, compared with PBS group, the OD_540_ of the diarrhea group was significantly increased (*P* < 0.01) upon infection by ETEC-K88, which indicated the neutrophilic granulocyte function was enhanced and the nonspecific immune balance was broken. Nevertheless, the OD_540_ was reduced in PHSP_(hp)_ and GLSP_(hp)_-treated mice serum. Thus, PHSP_(hp)_ and GLSP_(hp)_ may not only suppress the inflammatory response but also regulate nonspecific immunity.

**Table 2:** Effects of PHSP_(hp)_ and GLSP_(hp)_ on NBT activity (OD_540_). The activity of NBT was measured in mice sera from different groups upon ETEC-K88 infection^a^

	Nitroblue tetrazolium activity (OD_540_)
PBS	0.1284 ± 0.0082
Diarrhea	0.1940 ± 0.0122**
PHSP_(hp)_	0.1757 ± 0.0165^#^
GLSP_(hp)_	0.1422 ± 0.0073^##^

a Data are present as mean ± SD (*n* = 3). *P < 0.05 *versus* the PBS group, and **P < 0.01 *versus* the PBS group. ^#^*P* < 0.05 *versus* the diarrhea group, and ^##^*P* < 0.01 *versus* the diarrhea group.

## Discussion

In this study, two sulphated polysaccharides, PHSP_(hp)_ and GLSP_(hp)_, were extracted from *P. haitanensis and G. lemaneiformis*, respectively, by high pressure treatment and ethanol precipitation. MTT assay showed that PHSP_(hp)_ and GLSP_(hp)_ were not cytotoxic to the normal spleen lymphocytes of mouse (*P* > 0.05). The infrared spectrum results showed that hot water treatment and ethanol precipitation did not change the active ingredients of these polysaccharides. PHSP_(hp)_ and GLSP_(hp)_ have absorption peaks at 1030 cm^−1^ and 890 cm^−1^, indicating they are typical agar type polysaccharides.^31–33^ The infrared spectrogram data also shows some important functional groups of the polysaccharides. The absorption peaks at approximately 3340 cm^−1^, 2920 cm^−1^ and 1640 cm^−1^ represent –OH, C–H and C=C functional groups, respectively. In addition, the peak at about 1210 cm^−1^ was attributed to the sulphated ester groups, and the peak at about 1020 cm^−1^ corresponds to galactan backbone. However, altered physicochemical properties were also observed. For example, PHSP_(hp)_ and GLSP_(hp)_ showed more loose structure compared with polysaccharides extracted by hot water, as monitored by SEM. This result was consistent with previous finding that the changes of viscosity upon high pressure treatments and ethanol precipitation.^27^ The sulphated polysaccharides extracted from *P. haitanensis and G. lemaneiformis* showed average molecular weight of 2960 kDa and 152 kDa,^20,21^ with viscosity of 1.76 dL g^−1^ and 0.63 dL g^−1^, respectively, while the viscosity of PHSP_(hp)_ and GLSP_(hp)_ were 0.17 dL g^−1^ and 0.10 dL g^−1^, respectively. Decreased molecular weight and viscosity of PHSP_(hp)_ and GLSP_(hp)_ that underwent high pressure treatment may be beneficial to the digestion and absorption in the intestine of mice.

Macrophages are immune cells that actively participate in the inflammatory immune response by releasing inflammatory factors and pro-inflammatory cytokines.^34^ During inflammation, activated macrophages can secrete nitric oxide (NO) and many pro-inflammatory cytokines, such as IL-6, TNF-α and IL-1β.^35^ PHSP was shown to significantly increase the levels of TNF-α, IL-6, IL-10 and iNOS/NO in RAW264.7 cells.^27^ In this study, the immune regulation by PHSP_(hp)_ and GLSP_(hp)_ were demonstrated *in vitro*, including induced RAW264.7 cell migration, enhanced wound healing, as well as increased secretion of TNF-α and IL-6. The phagocytosis of pathogens by macrophages plays an important role in immune regulation, which involves the migration and adhesion of macrophages. PHSP_(hp)_ and GLSP_(hp)_ may act as chemokines to recruit macrophages and induce pathogen phagocytosis. Upon macrophage recruitment, they migrate to pathogens and sites of inflammation, and eventually exert their antibacterial effects.^23^

Bacteria intraperitoneal injection of has been a classic way to induce intestinal infection.^36,37^ To examine the anti-bacterial diarrhea activity of PHSP_(hp)_ and GLSP_(hp)_, BALB/c mice were infected with ETEC-K88 (LD_50_ = 4 × 10^8^ CFU) and then orally supplied with PHSP_(hp)_ and GLSP_(hp)_. The infected mice showed severe diarrhea, piloerection, lethargy and weight loss. However, PHSP_(hp)_ and GLSP_(hp)_ not only reduced the total amount of mouse diarrheal stools, but also prevented the loss of weight. Moreover, the levels of pro-inflammatory factors and IgA were significantly decreased (*P* < 0.05) upon PHSP_(hp)_ and GLSP_(hp)_ treatment. The flow cytometry results showed that B cells subpopulation was significantly increased after infection, which suggested that toxin antigen-attacked mice stimulate B cells to produce antibodies to engulf pathogens. This is consistent with high IgA content in the diarrhea group. And decreased IgA levels in PHSP_(hp)_ and GLSP_(hp)_ treated mice may resulted from the reduced B cell population. The reduction of pro-inflammatory factors, IgA, and B cell population suggested that PHSP_(hp)_ and GLSP_(hp)_ show anti-diarrhea activity through specific immune regulation in mice.

The neutrophil activity was considered a nonspecific immune parameter and determined by the NBT assay.^25^ The current study indicated that PHSP_(hp)_ and GLSP_(hp)_ reduced NBT level in ETEC-K88 infected BALB/c mice. Sulphated polysaccharides may stimulate the microbicidal activity of polymorphonuclear leukocytes and leucocytosis, which involves different nonspecific immune regulation mechanisms.^25^ The further effects of PHSP_(hp)_ and GLSP_(hp)_ on these leukocytes remains to be explored later.

Binding of toxins to ganglioside (GM) is the first step in toxins-induced diarrhea, which has become an attractive drug developing target for the treatment and prophylaxis of cholera toxins.^38^ Many studies have shown that galactose analogues interfere with the binding of toxin to GM1.^38^ The basic constituents of sulphated polysaccharides extracted from *P. haitanensis* and *G. lemaneiformis* are sulphated d-galactose monomers. PHSP_(hp)_ and GLSP_(hp)_ can alleviate ETEC-K88 bacteria diarrhea. This may be due to that the galactose monomers of sulphated polysaccharide block the toxin binding site and interfere with the binding of LT (heat-labile toxin) toxin to GM1. ETEC subtype STbP is involved in the induction of secretary diarrhea in animals, including humans. This toxin binds to sulphatide (3′-sulphogalactosyl-ceramide) receptors containing regions distributed on the intestinal epithelium cell wall.^39^ Inhibition of STbP binding to its receptor was achieved by type λ-carrageenan, a polymer extracted from seaweed and consisting of sulphated galactose units.^40^ It is speculated that PHSP_(hp)_ and GLSP_(hp)_ could alleviate diarrhea by mimicking this toxin to compete with the receptor.

Studies have reported that ganglioside receptors exhibit high affinity for carbohydrates, because toxins bind to ganglioside receptors in host epithelial cells in the form of glycoproteins and exhibit greater affinity for GM1.^41^ However, the galactoside acted as a high-affinity inhibitor of toxins-GM1 interaction. PHSP_(hp)_ and GLSP_(hp)_ comprises high-molecular-weight sulfated galactans, with high sugar content; galactose accounts for the majority of the sugars. It may be able to explain the interaction of PHSP_(hp)_ and GLSP_(hp)_ with GM1, preventing toxins binding, which interferes in the key step of transport of the toxin into the enterocytes.

## Conclusions

In this study, sulphated polysaccharides from *Porphyra haitanensis* (PHSP_(hp)_) and *Gracilaria lemaneiformis* (GLSP_(hp)_) were obtained by high pressure treatment plus ethanol precipitation, with the molecular weight of 165.45 kDa and 71.87 kDa, respectively. The wound healing and cell migration results indicated that PHSP_(hp)_ and GLSP_(hp)_ had immunomodulatory effects. They also alleviated diarrhea symptoms in mice *via* dietary intervention. Meanwhile, PHSP_(hp)_ and GLSP_(hp)_ inhibited the release of pro-inflammatory cytokines and IgA by reducing B cell population. In addition, they decreased nitroblue tetrazolium level in the ETEC-K88 infected mice. In summary, PHSP_(hp)_ and GLSP_(hp)_ were effectively against ETEC-K88-induced secretary diarrhea, probably by both specific and non-specific immunities. These findings may provide substantial support for the use of functional food as sulphated polysaccharides from red algae to alleviate diarrhea.

## Conflicts of interest

There are no conflicts to declare.

## Abbreviations

ELISA: Enzyme-linked immunosorbent assay
ETEC: Enterotoxigenic *Escherichia coli*
FT-IR: Fourier transformed infrared spectrometer
IFN-γ: Interferon-γ
IgA: Immunoglobulin A
IL-6: Interleukin-6
LD_50_: 50% lethal dose
LPS: Lipopolysaccharide
MCP-1: Monocyte chemotactic protein 1
NBT: Nitroblue tetrazolium
PHSP_(hp)_ and GLSP_(hp)_: Sulphated polysaccharides extracted from *Porphyra haitanensis* and *Gracilaria lemaneiformis* with high pressure
TNF-α: Tumor necrosis factor-α

## Supplementary Material
